# Addressing the silent threat: managing invasive *Candida* infections in hospitalized newborns

**DOI:** 10.3389/fped.2025.1613832

**Published:** 2025-07-17

**Authors:** Deshuang Zhang, Dongke Xie, Haokun Yuan, Na He, Wenbin Dong, Xiaoping Lei

**Affiliations:** ^1^ Department of Neonatology, Children’s Medical Center, The Affiliated Hospital of Southwest Medical University, Luzhou, Sichuan, China; ^2^ Department of Pediatrics, West China Second University Hospital, Sichuan University, Chengdu, Sichuan, China; ^3^ Department of Pediatric Surgery, Children’s Medical Center, The Affiliated Hospital of Southwest Medical University, Luzhou, Sichuan, China

**Keywords:** antifungal resistance, invasive *Candida* infections (ICIs), newborn infants, pathogens, prevention strategies, risk factors, treatments

## Abstract

Invasive fungal infections (IFIs) remain an important problem for hospitalized newborn infants receiving intensive care, given their substantial morbidity and mortality. *Candida* species (*Candida* spp.) are the major fungal pathogens, which cause the so-called invasive *Candida* infections (ICIs). Of these, *Candida albicans* is the most commonly isolated species, followed by *Candida parapsilosis*. Other identified *Candida* spp. include *Candida glabrata*, *Candida tropicalis*, *Candida krusei*, etc. However, an increasing shift in the epidemiology of ICIs worldwide has been described, non-*albicans Candida* (NAC) spp. ICIs pose a growing threat to neonates. Herein, we examine the epidemiology of *Candida* spp. infections, patterns of antifungal resistance, risk factors, prevention strategies, clinical outcomes, and treatment recommendations for ICIs in hospitalized newborn infants. This review aims to provide a thorough understanding of the current evidence on ICIs to better inform targeted prevention strategies and improved treatments to reduce neonatal morbidity and mortality.

## Introduction

Invasive fungal infections (IFIs), primarily invasive *Candida* infections (ICIs), remain an important problem for hospitalized infants receiving newborn critical care, as they are associated with substantial morbidity and mortality (1). Very low birth weight (VLBW) infants are at high risk for ICIs because of their immature immune systems, frequent exposure to invasive procedures and medical devices, use of broad-spectrum antimicrobial medications, prolonged parenteral nutrition and hospitalization, and postnatal corticosteroid exposure (1, 2). For VLBW infants, the incidence of ICIs across different centers ranges from 2.6%–13.2% and is even higher in extremely low birth weight (ELBW, birth weight <1,000 g) infants, from 6.6%–26.0% (3). The mortality rate remains above 25%, and nearly half of the survivors may develop significant long-term adverse outcomes, particularly neurodevelopmental impairment (NDI) (4, 5). Of note, there is a different pattern of ICIs among susceptible newborn infants in low- and middle-income countries (LMICs) compared to those in high-income countries (HICs). In HICs, ICIs are most commonly reported in ELBW infants, but reports from neonatal units in LMICs indicate ICIs occur in infants beyond this specific group (6–8). Larger infants with congenital malformations requiring surgery are increasingly affected by ICIs as a result of prolonged use of broad-spectrum antibiotics and increased duration of NICU stay in LMICs (6, 9–11).

Despite improvements in neonatal intensive care, advanced life support measures, and aggressive antifungal treatment, ICIs remain a persistent challenge, necessitating a thorough understanding of the current evidence on ICIs to better inform targeted prevention strategies and improved treatments to reduce neonatal morbidity and mortality.

## Common pathogens in newborn infants

A positive culture of fungal organisms from blood, urine, cerebrospinal fluid (CSF), or other sterile body fluids collected using techniques to minimize contamination with surface-colonizing organisms remains the standard for the diagnosis of IFIs, and can further identify the specific species (12, 13). IFIs in neonates are predominantly caused by *Candida* species (*Candida* spp.), which cause the so-called ICIs. Other fungal pathogens, including the yeast Malassezia and molds such as Aspergillus spp. and Zygomycetes, rarely cause nosocomial and mucocutaneous infections in newborn infants (14–17). In this review, we will focus on ICIs.

*Candida* is a genus comprising more than 200 fungal species, but only a minority are pathogenic and cause infections in humans (13, 18). Although *Candida* spp. are usually part of the normal flora and live as commensal organisms on the skin and mucous membranes, such as the oral cavity, and respiratory, gastrointestinal, and genitourinary tracts, they can transform into pathogenic forms under certain conditions (19). In particular, these yeasts have a higher binding affinity for mucosal surfaces than for the skin (18). *Candida* is present on the mucocutaneous surfaces of 84%–88% of individuals, including both hospitalized patients and healthy adults (20). More importantly, *Candida* spp. can adhere to and colonize the non-living surfaces of medical devices such as indwelling catheters, endotracheal tubes, and implants, posing important infectious risks (18). *Candida albicans* shows a greater adherence capability compared to other *Candida* spp., partially accounting for its higher prevalence and stronger correlation with infections (21). The transition of *Candida* spp. from a commensal relationship to a pathogenic state is driven by the expression of multiple virulence determinants. Specifically, these mechanisms include the formation of biofilms, the secretion of hydrolytic enzymes (e.g., proteinases, phospholipases, and hemolysins), the ability to adhere to host tissues and medical devices, and the transition to pseudohyphal growth (22, 23). In addition, the antifungal resistance profile has a significant impact on the virulence of *Candida* spp (19, 24). Mohammadi et al. found, for example, that higher minimum inhibitory concentrations (MICs) of fluconazole for *Candida albicans* are correlated with increased biofilm formation, elevated phospholipase production, and enhanced hemolysin activity (25). Similarly, Nakamura-Vasconcelos et al. revealed a positive correlation between fluconazole resistance in *Candida glabrata* and both enhanced adherence efficiency and increased biofilm formation (26).

In the NICU, nearly 75% of infants are colonized with *Candida* spp. by one month of age, either from maternal vertical transmission or horizontal nosocomial spread (27). Infants vaginally delivered are more likely to be colonized with *Candida* at birth than those born via cesarean section (C-section) (28), and further evidence supports that vaginal delivery is an important risk factor for neonatal *Candida* colonization (29, 30). This may be attributed to a significant increase in vaginal *Candida* colonization during pregnancy, particularly in the third trimester, with reported rates reaching up to 69.2% (31). *Candida albicans* is the most common *Candida* strain causing vaginal colonization in pregnant women, and it can be transmitted to their neonates (32, 33). According to Ali et al., *Candida albicans* accounted for 67.8% of maternal vaginal colonization and 77.7% of preterm infant colonization among all *Candida* colonization cases (33). Neonatal colonization and onset may predispose infants, particularly preterm infants, to the development of ICIs. Ali et al. reported that ICIs were identified in 22.2% of colonized preterm infants (33). Infants developing ICIs within the first postnatal week are more likely to have vertical transmission of *Candida* from the mother, with an associated higher mortality rate than those who develop the disease after the first week (34).

Historically, *Candida albicans* has been the organism most commonly isolated in ICIs, followed by *Candida parapsilosis* (35). Other *Candida* spp. identified include *Candida glabrata*, *Candida tropicalis*, *Candida krusei*, *Candida auris*, and *Candida lusitaniae*, et al (1, 11, 13). Consistently, *Candida albicans* is also the most frequently isolated organism responsible for ICIs in HICs (35). However, an increasing shift in the epidemiology of ICIs worldwide has been described, NAC spp. ICIs are emerging as a growing threat among NICUs in LMICs (10, 11, 36). Among the NAC spp., *Candida parapsilosis*, *Candida glabrata*, and *Candida tropicalis* are the most common isolates*.* A series of multicenter epidemiological studies present a high prevalence of *Candida albicans* in Northern Europe (37), the United States (38), England (39), Canada (40), and Saudi Arabiaand (41), accounting for more than 50% of *Candida* isolates. *Candida parapsilosis* is usually the most common NAC spp., with a prevalence of more than 35% in Southern Europe (37), Australia (42), and South Africa (43). The highest proportions (22.2%–44.4%) of *Candida glabrata* were reported in studies that were conducted in the central part of India (44), while *Candida tropicalis* is widespread in Eastern and Southern India (11, 36). Certainly, we should be aware that the distribution of species is very heterogeneous between regions and even between centers within a region. In recent years, the new pathogen *Candida auris* has shown an increasing incidence and is responsible for NICU outbreaks in South Africa, India, Colombia, and Venezuela, et al (45). The common pathogenic *Candida* strains in NICUs and their special profile are presented in Table 1.

**Table 1:** Common pathogenic *Candida* strains of invasive *Candida* infections (ICIs) in neonatal intensive care units (NICUs**)**.

*Candida* spp.	Prevalence profile	Microbial features	Clinical consequence (Reference)	Species distribution globally (Reference)
*Candida albicans*	Most commonly isolated strains, but decreasing globally	Higher virulence than that of NAC spp.: strong biofilm formation, high levels of proteinase and melanin production; prone to form cross-species biofilms	Higher rates of end-organ damage and greater mortality (51)	Prevalence in Northern Europe (37), the United States (38), England (39), Canada (40), and Saudi Arabia (41)
*Candida parapsilosis*	Increasing as pathogens	Less virulent: thinner biofilms, low virulence enzyme levels, no true hyphae, yeast/pseudohyphal forms	A relatively lower mortality rate than that caused by other *Candida* spp. (54)	Prevalence in Southern Europe (37), Australia (42), and South Africa (43)
*Candida glabrata*	Increasing as pathogens	Haploid genome; Frequently resistant to fluconazole	Limited data is available on the neonatal population	Prevalence in the central part of India (44)
*Candida tropicalis*	Increasing as pathogens	The second most virulent *Candida* spp.; Increasing resistance to azoles	Poor prognosis and high mortality rate (67, 68)	Prevalence in Eastern and Southern India (11, 36)
*Candida auris*	Emerging with rapid global spread	Multidrug-resistant strains; Difficult to identify; Thermotolerance and osmotolerance	Poor prognosis and high mortality rate (45)	NICU outbreaks in South Africa, India, Colombia, and Venezuela, et al. (45)

The incidence of neonatal ICIs usually peaks during the second to third week of life. Oeser et al. reported the median age at diagnosis for different *Candida* spp. as follows: 11 days for *Candida albicans*, 18 days for *Candida parapsilosis*, 9 days for *Candida glabrata*, 20 days for *Candida tropicalis*, and 23 days for *Candida lusitaniae* (46). However, a prospective cohort study from Northern India (6), one of the LMICs where early-onset sepsis is more common (in contrast to HICs) (47, 48), shows that most of *Candida spp.* were isolated during the first week of life with proven fungal sepsis, much earlier than in HICs. It is also worth noting that *Candida albicans* is the predominant strain in vertical transmission. *Candida parapsilosis* ICI more commonly results from nosocomial transmission. It is the most common *Candida* spp. colonizing the hands of healthcare workers, with a prevalence nearing 60% (49, 50).

In general, Candida albicans is typically more virulent than NAC spp., causes greater end-organ damage, and has higher attributable mortality (51). Makled et al. report that *Candida albicans* exhibited the highest virulence, featuring strong biofilm formation and high levels of proteinase and melanin production (19). Biofilms can both initiate and prolong infections by serving as a protective niche that resists treatment, enabling cells to invade local tissues and create new foci of infection (21). Proteinases contribute to fungal pathogenesis by degrading host cell membrane proteins, promoting adhesion and tissue invasion, while also disrupting host defense mechanisms to evade antimicrobial responses (52). Melanin production helps *Candida* evade the immune system, reducing phagocyte effectiveness and altering responses to antifungals (53). In comparison, *Candida parapsilosis* is generally associated with a relatively lower mortality rate than other *Candida* spp. A prospective observational study of ICIs in NICUs reported mortality rates of 39.5% for *Candida albicans* and 11.1% for *Candida parapsilosis* (54). *Candida parapsilosis* is less virulent, such as thinner and less structured biofilms, lower levels of virulence-associated enzymes, and the absence of true hyphal formation, existing instead in either a yeast phase or in a pseudohyphal form (55, 56). In addition, this species tends to develop biofilms on central venous catheters (CVCs) and other medical implants. It grows rapidly in parenteral nutrition with high glucose and lipid concentrations. Parenteral nutrition provides a medium that promotes *Candida parapsilosis* biofilm formation (49, 56, 57), making it more challenging to eradicate (58). Another concern is that *Candida* spp., particularly *Candida albicans*, are known for their association with various bacterial spp. in the formation of cross-species biofilms (59). Research has shown that *Candida albicans* frequently co-occurs in biofilms with a variety of bacteria, including *Staphylococcus* species, *Pseudomonas aeruginosa*, *Enterococcus faecalis*, etc. In the neonatal population, co-infection with *Candida albicans* and *Staphylococcus aureus* is frequently observed (21). This multi-species infection form of *Candida albicans* may be another important factor contributing to significant morbidity and mortality.

*Candida glabrata*, unlike *Candida albicans* and many other NAC spp., has a haploid genome, a key distinguishing genetic feature (60). It is frequently resistant to fluconazole, primarily due to the overexpression of efflux pump genes, particularly *CDR1*, mediated by the transcription factor *PDR1*, as well as mutations or upregulation of the *ERG11* gene, which encodes the target of azoles (61, 62). This resistance mechanism confers a competitive advantage to *Candida glabrata* in clinical settings where fluconazole is widely used, either for prophylaxis or treatment (57). In a clinical study, isolates of *Candida glabrata* were found more frequently in preterm infants with a higher gestational age (*Candida glabrata*: 30 weeks, *Candida albicans*: 26 weeks, *Candida parapsilosis*: 27 weeks) and birth weight (*Candida glabrata*: 1,442 g, *Candida albicans*: 931 g, *Candida parapsilosis*: 965 g) compared to those infected with other *Candida* spp (63).

*Candida tropicalis* is increasingly becoming an important pathogen in NICUs (64). In a recent systematic review and meta-analysis of neonatal candidiasis, investigators found that among a total of 402 *Candida* isolates, 9.5% were identified as *Candida tropicalis*, ranking third after *Candida albicans* and *Candida parapsilosis* (41). Genetically, this species is most similar to *Candida albicans* (65). It is widely regarded as the second most virulent *Candida* spp., surpassed only by *Candida albicans* (66). Among the NAC spp., *Candida tropicalis* generally has a high mortality and a poor prognosis and is classified as a high-priority pathogenic fungus by the WHO (67, 68). Another worrying feature of *Candida tropicalis* is the increasing rate of resistance to azoles. There are reports of resistance rates of around 15%–20%, compared to the previously observed rate of around 7% (68). However, given the limited availability of high-quality neonatal-specific data, further research is urgently needed to better understand the epidemiology, host-pathogen interactions, and resistance patterns of *Candida tropicalis* in neonates to better define the clinical impact in this vulnerable population.

*Candida auris* is an emerging, multidrug-resistant species that poses a significant and growing global public health threat due to its rapid worldwide spread (69, 70). It is frequently resistant to fluconazole, with variable resistance patterns to amphotericin B and echinocandins. Specifically, in general, 60%–90% of *Candida auris* strains are resistant to fluconazole, 10%–30% have high MICs for amphotericin B, and up to 5% are resistant to echinocandins (71). A more recent systematic review of 24 studies involving 476 neonates revealed a higher prevalence of antifungal resistance: 97% of cases were resistant to fluconazole, and 67% to amphotericin B (45). *Candida auris* has an unprecedented ability to spread rapidly in healthcare settings, not only through direct patient-to-patient transmission but also via contaminated medical devices such as thermometers. This strain can persist on environmental surfaces and equipment for long periods due to the formation of “dry” biofilms, rendering it thermotolerant and osmotolerant (72). Many commonly used hospital disinfectants are ineffective against it. Although initially identified in 2009 as a rare pathogen (73), *Candida auris* has emerged as a frequent cause of outbreaks in NICUs in many countries, primarily in LMICs (13, 45). *Candida auris* ICI has a mortality as high as 42% (45). Another concern is that *Candida auris* is challenging to identify and may be misidentified as *Candida haemulonii*, another emerging multidrug-resistant fungus. Such difficulties complicate its management and heighten the health threat (45, 74).

## Antifungal resistance patterns of *Candida* spp

As is known, there is considerable variability among different geographic regions, healthcare centers, and even individual units of *Candida* spp. that cause ICIs (75), and each *Candida* spp. poses a unique challenge to antifungal susceptibility profiles. The resistance status of *Candida* spp. is determined on the basis of clinical breakpoints (CBPs). CBPs are determined taking into account pharmacokinetic/pharmacodynamic parameters, relationships between clinical outcomes and MICs, and distributions of MIC values in wild-type fungal isolates (76). The Clinical and Laboratory Standards Institute (CLSI) and European Committee on Antimicrobial Susceptibility Testing (EUCAST) methods for susceptibility testing of yeasts are standardized and reproducible methods for susceptibility testing of fungi (77). If no MIC breakpoint is established, the epidemiological cutoff value (ECV) can be used (Table 2), which is based on an examination of the distribution of MIC values within a species.

**Table 2:** Clinical breakpoints for the most commonly used antifungal agents against common *Candida* spp. in neonates.

*Candida* spp.	Antifungal agents	MIC breakpoint (μg/ml)				ECV (μg/ml)
		S	I	SDD	R	
*Candida albicans*	Fluconazole	≤2	-	4	≥8	2
	Micafungin	≤0.25	0.5	-	≥1	
	Amphotericin B^b^					
*Candida parapsilosis*	Fluconazole	≤2	-	4	≥8	1
	Micafungin	≤2	4	-	≥8	
	Amphotericin B^b^					
*Candida glabrata*	Fluconazole	-	-	≤32	≥ 64	2
	Micafungin	≤0.06	0.12	-	≥ 0.25	
	Amphotericin B^b^					
*Candida tropicalis*	Fluconazole	≤2	-	4	≥8	2
	Micafungin	≤0.25	0.5	-	≥1	
	Amphotericin B^b^					
*Candida krusei* ^a^	Fluconazole	IR	IR	IR	IR	2
	Micafungin	≤0.25	0.5	-	≥1	
	Amphotericin B^b^					

Abbreviations: S, susceptible; I, intermediate; SDD, susceptible dose-dependent; R, resistant; MIC, minimum inhibitory concentration; IR, intrinsically resistant; ECV, epidemiological cutoff value.

^a^ *Candida krusei* is assumed to be intrinsically resistant to fluconazole, its MIC diameters should not be interpreted using this scale;—There are no sufficient data to establish MIC breakpoints.

^b^ No susceptibility test interpretive criteria are established for Amphotericin B; ECV can be used as a reference.

In general, antifungal-resistant *Candida* spp. remain uncommon in HICs. However, over the past decade, an increasing proportion of resistant *Candida* spp., particularly *Candida parapsilosis*, *Candida krusei*, and *Candida auris*, have emerged in LMICs. NeoOBS data from LMICs reported that among *Candida* spp., the overall resistance profile showed that 40% were resistant to fluconazole, 18% were resistant to amphotericin B, but there was no resistance to micafungin (10). Even more, a recent study from Eastern India revealed alarming resistance rates of fluconazole and amphotericin B among *Candida* spp., particularly NAC spp., reaching up to 51% and 35%, respectively (11). Herein, we present the 3 most commonly used drugs against the most common *Candida* spp. in neonates (Table 3).

**Table 3:** Antifungal resistance pattern of the different *Candida* spp. in neonates.

*Candida* spp.	Antifungal resistance pattern, Crude Sensitivity rate (Reference)		
	Fluconazole	Amphotericin B	Micafungin
*Candida albicans*	Uncommon	None	None
	More than 90% (10, 78)	Nearly 100% (10, 78)	Nearly 100% (10, 78)
*Candida parapsilosis*	Common in LMICs, especially in South Africa	Emerging in LMICs	None
		More than 90% (10)	Nearly 100% (10, 78)
	Decreased to around 40% (10, 79, 80)		
*Candida glabrata*	Common in LMICs, especially in India	None	None
		Nearly 100% (10, 78, 82)	Nearly 100% (10, 78)
	Decreased to less than 20% (6, 82)		
*Candida tropicalis*	Common in LMICs, especially in India	Emerging in LMICs, especially in India	None (Limited data)
			Nearly 100% (10, 78)
	Decreased to less than 60% (36)	Decreased to less than 90% (36)	
*Candida krusei*	Intrinsically resistant	Common in LMICs	None (Scarce data)
	Nearly 0% (82, 84)	Decreased to 60% (82, 85)	Nearly 100% (85)
*Candida auris*	`Frequent in LMICs	Frequent in LMICs	Uncommon (Scarce data)
	Decreased to less than10%–20% (10, 45)	Decreased to less than 20% (10)	Nearly 95% (71)

At present, resistance to antifungal agents in *Candida albicans* is still uncommon, although individual isolates may not conform to this general pattern. In a secondary analysis of ELBW infants with ICIs from the NICHD-Neonatal Research Network (NRN) study of HICs, 308 isolates were obtained from 110 infants, of which only two *Candida albicans* (2/184, susceptibility rate 98.9%) were resistant to fluconazole (78). The NeoOBS substudy of invasive candidiasis in LMICs found that 90.5% (38/42) of *Candida albicans* isolates were sensitive to fluconazole (10). The antifungal sensitivity to amphotericin B and micafungin was 100% (10, 78).

Resistance of *Candida parapsilosis* to antifungals is gradually increasing in LMICs. The NICHD-NRN data from HICs showed that none of the 107 *Candida parapsilosis* isolates were resistant to fluconazole, amphotericin B, or micafungin (78). The NeoOBS data from LMICs reported that the susceptibility rate was only 40.6% (13/32) for fluconazole, 93.1% (27/29) for amphotericin B, but also 100% for micafungin (10). Other South Africa series reported similar data; fluconazole resistance rates were 53%–55% (79, 80).

*Candida glabrata* is more susceptible to developing fluconazole resistance than other *Candida* spp (81). It is thought that azole resistance has increased so much in *Candida glabrata* isolates that it is difficult to rely on these agents for therapy in the absence of susceptibility testing (75). Data from HICs with a low rate indicated that one of the *Candida glabrata* isolates (1/9) was resistant to fluconazole, but no isolates were resistant to amphotericin B or micafungin (78). In a study from India, the susceptibility rate of *Candida* *glabrata* to fluconazole was only 26.3% (5/19), but susceptibility to amphotericin B was 100% (19/19) (82). In another study from India, the fluconazole resistance rate was found to be 87.5% (6). In a previous observational study conducted by our team, we found that the overall susceptibility rate of this isolate to fluconazole decreased significantly (from 85%–40%) after prophylactic fluconazole, although there were no resistant isolates (1). The main reason for this could be related to the frequent occurrence of *Candida glabrata*. The overall susceptibility rate of *Candida* spp. to amphotericin B was consistently 100%.

*Candida tropicalis*, which is prevalent in India, is more often resistant to fluconazole and/or amphotericin B. The resistance rates of this species to fluconazole and amphotericin B were as high as 44% and 14.2%, respectively (36). The susceptibility to micafungin was also 100%, but only limited data were available (10, 78).

*Candida krusei*, a potentially multidrug-resistant opportunistic species, is emerging in LMICs. It is intrinsically resistant to fluconazole and can also rapidly acquire resistance to other antifungal agents (82, 83). Among the 1,075 *Candida krusei* isolates tested, the crude percentage of resistance to fluconazole was 96.6%, according to epidemiological data (84). Some data from India indicated that the susceptibility rates to amphotericin B have decreased to 60%–86% (82, 85), and scarce data found no resistance to micafungin (85).

*Candida auris*, a rapidly emerging multidrug-resistant causative pathogen, usually causes outbreaks in LMICs. NeoOBS data from LMICs showed high resistance to fluconazole (15/17, 88%) and amphotericin B (11/13, 85%) in *Candida auris* isolates, but no resistance to micafungin; however, many isolates were not tested (10).

Taken together, in neonates, *Candida* spp. especially NAC spp. are gradually developing resistance to antifungal agents, particularly fluconazole (the most commonly used antifungal agent in neonates), followed by amphotericin B. However, current studies are predominantly retrospective and thus possess inherent limitations; susceptibility testing for *Candida* spp. remains sparse and restricted, leading to an overall low level of evidence. These data only provide an approximate overview of the antifungal resistance patterns of the different *Candida* spp. in hospitalized newborn infants. There is an urgent need for large-scale, prospective studies to establish a robust framework for susceptibility testing of *Candida* spp. in neonatal units. This will allow a better understanding of these patterns and help in formulating strategies for the use of appropriate antifungal agents against ICIs in neonates.

## Potential risk factors

**Figure 1:**
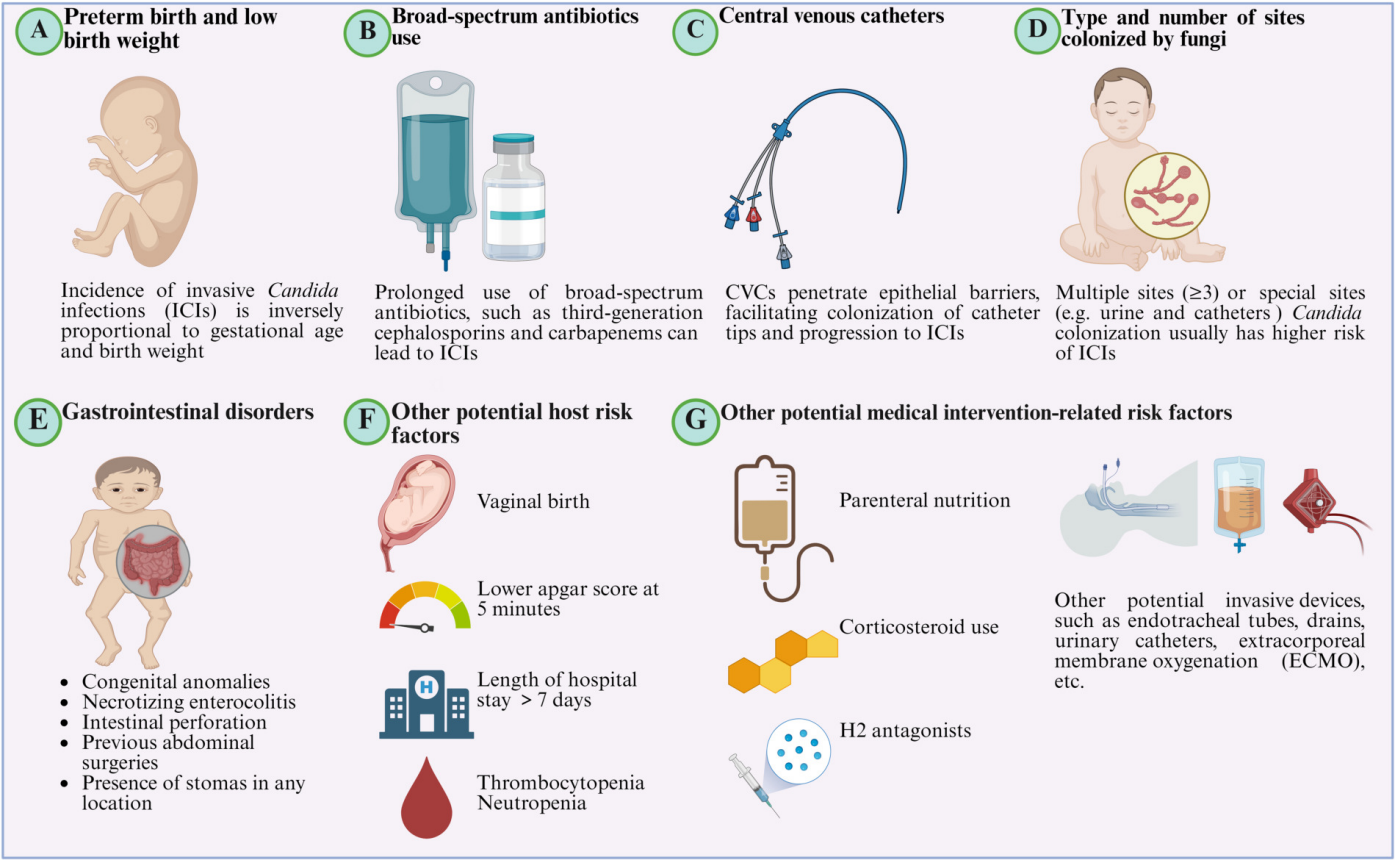
Potential risk factors for the incidence of invasive *Candida* infections **(**ICIs). **(A)** Preterm birth and low birth weight. Gestational age and birth weight are key risk factors for ICIs, with incidence inversely related to both. **(B)** Broad-spectrum antibiotics use. Prolonged use of broad-spectrum antibiotics, especially third-generation cephalosporins (TGCs) and carbapenems, suppresses normal flora, allowing fungi to colonize mucosal niches and facilitating subsequent invasion and spread. **(C)** Central Venous Catheters (CVCs). CVCs penetrate epithelial barriers, facilitating colonization of catheter tips and progression to ICIs. **(D)** Type and number of sites colonized by fungi. Multiple sites (≥3) or special sites (e.g., urine, catheters) colonized by *Candida* spp. are associated with a higher risk of ICIs. **(E)** Gastrointestinal disorders. Gastrointestinal disorders, including congenital anomalies, as well as necrotizing enterocolitis (NEC), intestinal perforation, previous abdominal surgeries, and the presence of stomas in any location, predispose cases to ICIs. **(F)** Other potential risk factors. Other potential host risk factors include vaginal birth, lower Apgar score at 5 min, length of NICU stay >7 days, thrombocytopenia, neutropenia, etc. **(G)** Other potential medical intervention-related risk factors. Related risk factors also include parenteral nutrition, corticosteroid use, H2 antagonists, and other potential invasive devices, such as endotracheal tubes, drains, urinary catheters, extracorporeal membrane oxygenation (ECMO), etc. (Created in BioRender.com).

Hospitalized newborn infants are at high risk for ICIs due to host and environmental factors (Figure 1, Table 4).

**Table 4:** Potential risk factors for invasive *Candida* infections (ICIs) in neonates.

Risk factors	Details (Evidence reference)	Potential mechanisms of action
Preterm birth and low birth weight	Incidence of ICIs is inversely proportional to gestational age and birth weight (86, 87)	Poor epithelial barrier easily disrupted → extracellular matrix exposure and serum leakage → allow *Candida* spp. adhesion;
		Lower vernix caseosa (protective biofilm) → reduce the ability to inhibit fungi;
		Reduced antimicrobial peptides, decreased phagocytes, impaired neutrophil function, and compromised pathogen recognition → reduced ability to inhibit fungi;
		Immature adaptive immune response and prone to immune tolerance → increase susceptibility to ICIs
Broad-spectrum antibiotics use	Prolonged use of broad-spectrum antibiotics is significantly associated with ICIs (86, 99, 100)	Broad-spectrum antibiotics → suppress the normal flora → allow fungi to occupy mucoepithelial niches → facilitate fungi invasion and spread
Central venous catheters (CVCs)	An essential part and a significant risk factor for ICIs in NICUs (12, 102, 103)	CVCs use → penetrate epithelial barriers → allow *Candida* enter bloodstream and colonize catheter tips → progress to ICIs
Type and number of sites colonized by fungi	Multiple sites or special sites colonized by fungi correlate with the risk of progression to ICIs (102, 106, 107)	*Candida albicans* has greater pathogenicity and adaptability → the most common colonized fungal spp. in multiple sites → progress to ICIs;
		Urine and catheters → high-risk colonization sites → progress to ICIs
Other potential factors	Gastrointestinal disease predispose cases to ICIs (9)	Compromised intestinal barrier → facilitate translocation and colonization of *Candida* → progress to ICIs
Gastrointestinal disease		
Parenteral nutrition	A predisposing factor for ICIs, independent of the use of CVCs (117)	Parenteral nutrition → ideal medium for *Candida* growth → progress to ICIs;
		Contamination during the preparation → *Candida* outbreaks
Corticosteroids	Increase ICIs risk in vulnerable premature population (118)	Corticosteroids → reduce circulating lymphocyte subpopulations, inhibit cytokine responses, and impair cell-mediated immunity → increase risk of ICIs
H2 antagonists	Increase ICIs risk in vulnerable premature population (117)	Weaken gastric acid barrier → promote *Candida* growth;
		Increase production of pro-inflammatory cytokines, decrease neutrophil activity, and reduce immunological responses to infection → increase ICIs risk
Other invasive devices, like endotracheal tubes, drains, urinary catheters, extracorporeal membrane oxygenation (ECMO), etc	Linked to an increased risk of ICIs (9, 122, 123)	Serve as portals of entry and adhesion for *Candida* → progress to ICIs

### Preterm birth and low birth weight

Gestational age and birth weight are the most important risk factors for the development of ICIs (86, 87). A large multicenter cohort study of 4,579 ELBW infants revealed significant associations between birth weight/gestational age and the risk of ICIs. Infants with a birth weight of 400–750 g or gestational age of 22–25 weeks had a 3–5 -fold increased risk compared to those weighing 751–1,000 g or 26–28 weeks (11%–10% vs. 3%–2%, 95% CI 2.47–4.19) (86). A large retrospective study of 530,162 infants weighing greater than 1,500 g from 305 NICUs reported a 0.06% ICI incidence (87), indicating that ICIs are rarely observed in neonates born after 32 weeks of gestation and/or with a birth weight greater than 1,500 g, especially in HICs.

The epithelial barrier serves as the first line of defense against exogenous pathogens. In term infants, this barrier is well developed, whereas, in most immature newborns, such as those born preterm or with low birth weight, it is poorly developed and can be easily disrupted (88, 89). Such disruption can lead to exposure of the extracellular matrix and serum leakage into the mucosa. This creates a favorable environment for *Candida* spp. to establish adhesion as pathogen-derived adhesins form a bridge to the host epithelial surface (90). Furthermore, preterm or low birth weight infants usually have a lower amount of vernix caseosa, a protective biofilm that forms in the hair follicles during the last trimester of pregnancy (89, 91). Vernix caseosa has good antimicrobial properties, is enriched with lysozyme, lactoferrin, and antimicrobial peptides, and has been shown to inhibit common bacterial and fungal pathogens (89, 92). Preterm infants have a range of differences in innate immunity, including reduced levels of antimicrobial peptides, decreased numbers of phagocytes (neutrophils and monocytes), altered neutrophil function, and compromised pathogen recognition due to immature functionality of pattern recognition receptors (PRRs) (91). These immune differences in preterm newborns increase the risk of infection, in particular for ICIs. The adaptive immune response in preterm infants remains immature and is prone to immune tolerance. In neonates, T cells are primarily naive and exhibit limited response to microbial antigens due to a lack of prior exposure during gestation (93). Additionally, neonatal CD4T cells have an intrinsic reduced ability to differentiate into Th17 cells, which play a critical role in controlling *Candida* proliferation (94). In term infants, antigen-presenting cells (APCs) produce elevated levels of Th17-polarizing cytokines, such as IL-1β and IL-23 (95), which are essential for driving the differentiation of naive CD4T cells into Th17 cells. In contrast, dendritic cells and monocytes from preterm infants, especially those born before 29 weeks of gestation (96, 97), have significantly reduced production of these cytokines and impaired antigen presentation, which further increases their susceptibility to ICIs.

Of particular note, in LMICs, especially in some regions of India, larger infants born after 32 weeks' gestation, with a birth weight ≥1,500 g, or even term infants, account for a larger proportion of ICIs (6, 11). In a tertiary neonatal unit of these regions, a large number of higher birth weight infants are usually admitted after being referred from peripheral units for surgical or cardiac morbidities. Most of these infants have a history of prior exposure to broad-spectrum antibiotics.

### Broad-spectrum antibiotics use

In NICUs, broad-spectrum antibiotics such as third-generation cephalosporins (TGCs) and carbapenems are frequently used and sometimes for long durations. Antibiotic exposure poses a risk for ICIs, especially those caused by *Candida* spp., as they suppress the normal flora, thereby allowing fungi to occupy mucoepithelial niches and facilitating subsequent invasion and spread (98). A multicenter study involving 3,702 ELBW infants found that the average duration of TGC use was significantly associated with the occurrence of ICIs, with a correlation coefficient of 0.67 (*P* = 0.017) (99). Similarly, another study of 4,579 ELBW infants revealed that the use of TGCs on hospital day 3 significantly increased the risk of subsequent ICIs compared to other antibiotics [15.3% vs. 5.6%, odds ratio (OR) 1.77, 95% CI 1.31–2.38] (86). Hou et al. pointed out that a 10% increase in antibiotic use leads to a 71% increase in the risk of ICIs (OR 1.71, 95% CI 1.41–2.08). Each additional day of antibiotic exposure increases the risk of IFIs by 13% (OR 1.13, 95% CI 1.06–1.20). The use of TGCs and carbapenems was associated with a 17% (95% CI 1.04–1.33) or 18% (95% CI 1.06–1.30) increased risk of ICIs, respectively, for each additional day of exposure (100). Conversely, Aliaga et al. reported that a 10% reduction in the use of broad-spectrum antibiotics was associated with a 3%–7% decrease in ICI episodes for VLBW infants (3).

### Central venous catheters (CVCs)

CVCs are an essential part of the care of VLBW infants during their stay in NICUs. The use of CVCs is a well-documented and significant risk factor for ICIs in neonates. CVCs penetrate epithelial barriers, allowing *Candida* to enter the bloodstream and preferentially colonize the catheter tips, which is often associated with the normal resident skin flora at the insertion site (101).

Colonization with *Candida* spp. is an essential first step in the pathogenesis of ICIs, and CVC colonization represents a significant risk factor for progression to invasive disease. A previous study of 689 VLBW infants in the NICU found that infants with a *Candida*-colonized CVC had a nearly tenfold higher risk of progression to ICI than infants without a colonized CVC (OR 10.81, 95% CI 1.45–8.10) (102). Furthermore, CVCs facilitate the formation of *Candida* biofilms, which play an important role in the development of ICIs in neonates. In addition, a study reported that the risk of ICIs increases with each additional day that CVCs remain in place, with an OR of 1.06 per day of use (95% CI 1.02–1.10) (103). It is particularly crucial to remove CVCs at the earliest opportunity during ICIs when the CVC is suspected to be the source and its removal is feasible (12). The potential for drug-resistant biofilm formation highlights the importance of the timely removal of catheters, especially in ELBW infants with ICIs. Delayed removal has been associated with prolonged ICIs and increased risk of end-organ involvement, NDI, and higher mortality (86, 104). For example, Benjamin et al. observed that mortality and NDI rates were significantly higher in infants in whom catheter removal or replacement was delayed (more than one day after initiation of antifungal treatment), with an OR of 2.69 (95% CI 1.25–5.79), compared with those timely removal (86).

### Type and number of sites colonized by fungi

Some studies have shown that the type and number of sites colonized by fungi correlate with the risk of progression to ICIs in neonates. Mahieu et al. found no cases of ICIs in neonates colonized exclusively on the skin. However, in neonates with gastrointestinal colonization, the prevalence of ICIs was 16.6% and increased to 41.7% when both skin and gastrointestinal sites were colonized (105). A cohort of 201 VLBW infants colonized with *Candida* spp. identified colonization at multiple sites (≥3) as an independent risk factor for the development of ICIs during hospitalization (OR 6.15, 95% CI 2.40–7.69) (102). Subsequently, Manzoni et al. further reported a threefold increase in ICI incidence in VLBW infants colonized at more than three sites (106). One possible explanation for this finding is that *Candida albicans* is the most common fungal spp. in multiple sites colonized by fungi and has greater pathogenicity and adaptability (107). In addition, Manzoni et al. revealed that high-risk colonization sites such as urine and catheters were associated with a fourfold higher risk of ICIs than low-risk sites like skin, nasopharyngeal secretions, and gastric aspirates (106).

### Other potential risk factors

Gastrointestinal disorders, including congenital anomalies such as gastroschisis, omphalocele, and duodenal or ileocolic atresia/stenosis, as well as necrotizing enterocolitis (NEC), intestinal perforation, previous abdominal surgeries, and the presence of stomas in any location, predispose cases to ICIs (9). This predisposition arises from a compromised intestinal barrier that facilitates the translocation of *Candida* spp. colonizing the gastrointestinal tract (108). Multiple studies have demonstrated a close correlation between gastrointestinal diseases and ICIs in neonates (109–111). Other potential host risk factors include prior *Candida* colonization (112), vaginal birth (40), lower Apgar score at 5 min (40), length of NICU stay >7 days (113), thrombocytopenia, platelet counts <50,000/mm^3^ (87), neutropenia, and neutrophil count <1,500/mm^3^ (114).

Parenteral nutrition is necessary for supporting the nutritional needs of VLBW infants in NICUs. Parenteral nutrition infusions, especially those with high glucose content and rich fat emulsions, provide an ideal medium for *Candida* growth and promote the formation of drug-resistant biofilms on catheters (49, 115). Moreover, contamination during the preparation of parenteral nutrition solutions continues to be a potential contributing factor to *Candida* outbreaks in NICUs (116). It is also important to know that parenteral nutrition serves as a predisposing factor for ICIs, independent of the use of CVCs (117). Corticosteroids are commonly used in very premature infants to reduce the need for ventilatory support and its duration, as well as to reduce pulmonary morbidity (118). A recent survey of 397 NICUs across Europe found that the majority of these units administer corticosteroids in the second or third week of life. This practice aims to facilitate extubation and/or prevent bronchopulmonary dysplasia (BPD) in high-risk infants, regardless of the type of ventilatory support (119). It is well documented that corticosteroids can reduce circulating lymphocyte subpopulations, inhibit cytokine responses, and impair cell-mediated immunity in preterm infants, and thus increase the risk of ICIs in this vulnerable population (118). H2 antagonists act by inhibiting gastric acid secretion, resulting in an elevated stomach pH. This weakened gastric acid barrier promotes the growth of Gram-negative bacteria and *Candida* spp., which can then spread through the gastrointestinal tract (120). Manzoni et al. found that each additional day of exposure to gastric acid inhibitors was associated with a 4.5% (95% CI 1.02–1.07) and 6.3% (95% CI 1.03–1.10) increased risk of fungal colonization and fungal infection, respectively, in preterm infants (120). In addition, H2 antagonists can increase the production of pro-inflammatory cytokines, decrease neutrophil activity, and reduce immunological responses to infection (121). A prospective cohort study including 2,847 infants across multiple NICUs found that the administration of H2 antagonists was associated with a 2.44-fold increased risk (95% CI 1.11–5.29) of developing candidemia in neonates. Other potential invasive devices, like endotracheal tubes, drains, and urinary catheters, similar to CVCs, serve as portals of entry and adhesion for *Candida* spp. and may contribute to nosocomial transmission (9). In an epidemiologic surveillance study, compared to CVCs and urinary catheters, endotracheal tubes were found to be linked to the highest risk [risk ratio (RR) 22.9, 95% CI 5.6–93.8] of developing nosocomial infection, including those caused by *Candida* spp (122)*.* This study also found that two catheters increased the relative risk for nosocomial infections by 2.6 times (95% CI 1.3–4.9), while the use of three catheters increased it by 3.6 times (95% CI 1.9–7.1). In a similar pattern, extracorporeal membrane oxygenation (ECMO) procedures may also contribute to the risk of acquired fungal infection, and *Candida* spp. was the most common pathogen. A greater number of ECMO cannula placement procedures were independently linked to an increased risk of acquired infection, including fungal infection during ECMO treatment [hazard ratio (HR) 2.13, 95% CI 1.22, 3.72] (123).

## Prevention strategies

Given the serious consequences of ICIs for survival and NDI in very premature and VLBW infants, proactive prevention emerges as a critical strategy (Table 5).

**Table 5:** Antifungal prophylaxis agents for invasive *Candida* infections (ICIs) in neonates.

Agents	Recommended dosing for VLBW infants (**Reference**)	Major concerns in using	Potential mechanisms of action	Evidence on efficacy
Fluconazole	3–6 mg/kg, twice weekly, orally or intravenously in NICUs with high rates (>10%) (12, 133)	Potential fluconazole-resistant fungal strains, elevation in the minimum inhibitory concentrations (MIC) of *Candida*	Disrupts the production of ergosterol, a fungal cell wall component, causing toxic lanosterol accumulation in the membrane	High-quality evidence
Nystatin	100,000 units, three times daily, orally (12, 133)	Potential damage to the delicate intestinal epithelium, and the risk of necrotizing enterocolitis (NEC)	Reduces fungal colonization density in the gut, lowering the risk of fungal dissemination into the bloodstream;	Moderate-quality evidence
			Binds to ergosterol in the fungal cell membrane, increasing permeability, causing intracellular leakage, and cell death	
Probiotics	Optimal strain selection, dosage, and duration are unknown	Probiotic sepsis	Targets virulence factors, produces active metabolites (e.g., bacteriocin), and regulates host immune response;	Unknown
			Competes with pathogens (bacteria, fungi)	
Bovine lactoferrin	100 mg (12, 133) or 150–300 mg/kg (162, 169, 170), daily, orally	Reduction in ICIs remains inconsistent	Broad-spectrum antimicrobial activities: disrupts cell membranes, inhibits adhesion, prevents biofilm, sequesters iron;	Moderate-quality evidence
			Promotes probiotic growth, stimulates enterocyte differentiation and proliferation, increases digestive enzyme expression	

### Antifungal prophylaxis

Antifungal prophylaxis has been proven to be effective in reducing fungal colonization and the incidence of ICIs, thereby reducing associated mortality and long-term disability. At birth, most VLBW infants have either no or minimal fungal colonization, creating a critical window for antifungal interventions. These drugs can effectively prevent initial colonization or suppress the growth and spread of yeast in infants already colonized. It is worth highlighting that without antifungal prophylaxis, *Candida* colonization can affect up to 60% of VLBW infants by their second to third week of life (124).

#### Fluconazole prophylaxis

Among antifungal agents, fluconazole (oral or intravenous), a member of the azole class, has emerged as the most commonly used prophylactic option for high-risk neonates. Fluconazole acts by selectively inhibiting cytochrome P450 enzymes, particularly 14α-demethylase, a critical enzyme in the synthesis of ergosterol, which is essential for the integrity of the fungal cell membrane (125). When ergosterol production is disrupted, toxic precursors such as lanosterol accumulate in the membrane and impair its structure and function. This leads to damage to the fungal cells and effectively suppresses the growth and reproduction of fungi.

Numerous RCTs and retrospective studies have shown fluconazole to be safe and effective in reducing the incidence of ICIs in VLBW or ELBW infants, although the effect on mortality reduction in these studies is inconsistent (1, 126–130). An earlier meta-analysis of 578 VLBW infants in the United States comparing fluconazole prophylaxis with placebo showed that fluconazole significantly reduced the likelihood of *Candida* colonization (OR 0.28, 95% CI 0.18–0.41) and ICIs (OR 0.20, 95% CI 0.08–0.51). However, there was no significant reduction in mortality (OR 0.68, 95% CI 0.40–1.13) in those receiving fluconazole (131). A recent meta-analysis of 1,635 VLBW infants found that fluconazole prophylaxis significantly reduced the rate of fungal colonization (RR 0.32, 95% CI 0.24–0.41), the incidence of ICIs (RR 0.37, 95% CI 0.21–0.65) and ICI-related mortality (RR 0.17, 95% CI 0.05–0.64) compared with the control group (132). The Infectious Diseases Society of America (IDSA) and ESCMID guidelines recommend 6 weeks of prophylactic fluconazole use (3–6 mg/kg, twice weekly, either orally or intravenously) for VLBW infants in NICUs where the incidence of ICIs exceeds 10% (12, 133). The decision between 3 mg/kg and 6 mg/kg fluconazole prophylaxis administered twice weekly should be guided by local MIC data and resistance patterns for ICIs (134).

A major concern is that fluconazole prophylaxis may promote the development of fluconazole-resistant fungal strains. Although the emergence of resistance is rare and remains controversial, sporadic reports indicate an increase in fluconazole-resistant strains and a slight elevation in the MICs of *Candida* among exposed preterm infants (14, 135–137). This finding was also corroborated in our previous study (1). There is evidence that *Candida* spp. may cooperate after exposure to a low dose of fluconazole, leading to a gradual increase in MICs and an expansion of the azole resistance spectrum (107, 138).

#### Oral nystatin

Nystatin is the most frequently used oral, non-absorbable agent for the prophylaxis of *Candida* infections (139). Non-absorbable antifungals, which are not absorbed systemically, aim to reduce the density of fungal colonization in the gastrointestinal tract and thus reduce the risk of fungal dissemination from the intestine into the bloodstream (133). In addition, nystatin binds to ergosterol in the fungal cell membrane, increasing membrane permeability and leading to leakage of intracellular components and eventual cell death (13). Although it has not been studied as extensively as fluconazole prophylaxis, several studies have shown that nystatin is also effective in reducing *Candida* colonization and ICIs (138, 140, 141). However, data on mortality associated with prophylactic nystatin remain limited.

In an RCT of 278 VLBW infants, prophylactic administration of nystatin significantly reduced the incidence of *Candida* colonization (11.7% vs. 42.9%) and ICIs (4.3% vs. 16.5%) compared to the control group, but no significant difference was found between the two groups in terms of deaths due to ICIs (1.1% vs. 3.3%) (138). A Cochrane meta-analysis of 1,800 VLBW infants evaluated the efficacy of oral non-absorbable antifungal prophylaxis, primarily nystatin, compared to placebo. The analysis showed a significant reduction in the incidence of ICIs (RR 0.20, 95% CI 0.14–0.27), but also no significant effect on mortality (RR 0.87, 95% CI 0.72–1.05) (142). Similarly, a recent systematic review and meta-analysis involving 1,750 VLBW infants showed that oral nystatin was associated with a significant reduction in *Candida* colonization (RR 0.34, 95% CI 0.24–0.48) and ICIs (RR 0.15, 95% CI 0.12–0.19) compared to the control group, with no significant difference in mortality observed between the two groups either (RR 0.87, 95% CI 0.64–1.18) (143).

A potential concern with the use of nystatin is the risk of inadvertent damage to the delicate intestinal epithelium of preterm infants, which may contribute to the development of NEC (133). An RCT of 80 VLBW infants compared the efficacy of oral nystatin with fluconazole prophylaxis (144). The study was terminated prematurely due to the unfavorable prognosis associated with nystatin. Despite being underpowered, the findings indicated a significantly higher mortality rate in the nystatin group compared to the fluconazole group (7.5% vs. 0.0%, six deaths in the nystatin group, four of which were related to NEC). Furthermore, oral administration of nystatin has its limitations due to the frequent dosing regimen and lack of suitability for infants with gastrointestinal problems, a condition commonly observed in very premature and VLBW infants. The IDSA recommends six weeks of treatment with oral nystatin at a dosage of 100,000 units administered three times daily as an alternative when fluconazole is not available or contraindicated due to resistance (12), which is consistent with the moderate recommendation of ESCMID (133).

### Probiotics

Probiotics are live bacterial organisms that, when administered in sufficient quantities, provide health benefits to the host through the modulation of the gut microbiota (145). The potential benefits of probiotic supplementation for VLBW infants have been an ongoing focus of numerous studies in the past two decades. Probiotics have been shown to have an antifungal effect by targeting the virulence factor of fungi, producing active metabolites, particularly bacteriocin, regulating the host immune response, etc (146). The gastrointestinal commensal microbiota could also compete with pathogenic organisms such as bacteria or fungi (146, 147). Meanwhile, the gastrointestinal tract is an important site for *Candida* colonization and a mucosal surface for translocation. Broad-spectrum antibiotics may lead to dysbiosis and increase the risk of ICIs; it follows that the reintroduction of commensal microbiota may reduce this risk; however, this has not been confirmed for hospitalized newborns in rigorous clinical trials.

In an RCT of 62 extremely preterm infants (born at <29^0/7^ weeks of gestation and weighing ≤1,000 g), Alshaikh et al. found that multi-strain probiotics significantly increased fecal levels of *Bifidobacterium* and *Lactobacillus*, while markedly reducing the abundance of *Candida* in the stool (148). Two prospective, randomized comparative studies were conducted in VLBW infants. In both cases, prophylactic supplementation of *Lactobacillus reuteri* and *Saccharomyces boulardii* showed similar efficacy to nystatin prophylaxis in reducing fungal colonization and ICIs (149, 150). However, a meta-analysis of eight RCTs found no significant benefit of probiotics in preventing ICIs (RR 0.89, 95% CI 0.44–1.78). Another meta-analysis of seven RCTs, which included 1,371 preterm infants administered with strains of *Bifidobacterium*, *Lactobacillus*, or a combination of both, demonstrated a reduced incidence of *Candida* colonization compared to infants who did not receive probiotics (RR 0.43, 95% CI 0.27–0.68). However, there was no statistically significant impact observed on the incidence of ICIs (RR 0.88, 95% CI 0.44–1.78) (151). Even more, in a multicenter-matched cohort study of 2,178 preterm infants from 392 NICUs, infants receiving probiotics had an elevated risk of ICIs relative to those not receiving probiotics (OR 2.23, 95% CI 1.29–3.85). However, the absolute difference in the incidence of *Candida* infection was relatively minor (1.0% in the probiotic group vs. 0.4% in the non-probiotic group) (152). To sum up, conflicting data presents its efficacy in preterm infants, which may be based on the massive heterogeneity of study protocols, such as the strain selection, dosage, and duration of administration, needing further and ongoing investigation.

Another concern regarding the administration of probiotics in preterm infants is the potential risk of triggering sepsis, also termed “probiotic sepsis”. Probiotic sepsis is severe and sometimes life-threatening. This complication is defined as positive cultures from blood or CSF that isolate the strain of the administered probiotics, along with clinical signs of infection (153). Although rare, several reports have documented individual cases or case series of sepsis associated with probiotic supplementation in preterm infants (154–156). The efficacy and safety of probiotic strains administered are therefore all potential barriers to the use of prophylactic probiotics in preterm infants. Of particular importance is the American Academy of Pediatrics (AAP) statement opposing the routine use of probiotics, as well as the regulatory restrictions imposed by the US Food and Drug Administration (FDA) on their use in preterm infants, especially ELBW infants, the highest risk population for ICIs.

### Bovine lactoferrin (BLfcin)

Lactoferrin (Lfcin), a mammalian glycoprotein found in milk and belonging to the transferrin family, is an important bioactive component of whey protein and accounts for about 10%–20% of the total protein content of milk (157). It plays a key role in the innate immune response of mammals to infections. Multiple studies have demonstrated its broad spectrum of antimicrobial properties, including mechanisms such as disruption of microbial cell membranes, inhibition of microbial adhesion to host cells, prevention of biofilm formation, and sequestration of iron (158, 159). In addition, Lfcin facilitates the growth of probiotic bacteria, stimulates differentiation and proliferation of enterocytes, increases the expression of digestive enzymes, and shows direct immunomodulatory and anti-inflammatory effects in the gut (160). Very premature infants often receive little or no milk in the early postnatal period, resulting in a low intake of Lfcin. This can be exacerbated by delays in the introduction of enteral feeding. To address this immunodeficiency, enteral supplementation of bovine Lfcin (bLfcin) has been proposed as a simple strategy (161). BLfcin shares approximately 70% homology to human Lfcin (hLfcin) but has a higher antimicrobial activity due to the different three-dimensional structures (162, 163). Specifically, bLfcin in solution forms a *β*-sheet conformation containing a group of aligned hydrophobic residues that are well suited for interactions with biological membranes, whereas hLfcin in solution forms a coiled structure that lacks these aligned residues and therefore has weaker interactions with target cells (164–166).

A secondary analysis of a multicenter RCT involving 472 preterm infants in Italy found a significantly lower incidence of ICIs in VLBW infants receiving bLfcin alone (0.7%) or in combination with *Lactobacillus rhamnosus* GG (2.0%) compared to the placebo group (7.7%). However, the fungal colonization rates were comparable across the three groups (17.6%, 16.6%, and 18.5%, respectively) (167). Nonetheless, a UK-based RCT involving 2,203 infants born before 32 weeks of gestation found no significant difference in the incidence of late-onset sepsis (LOS), including ICIs, between the infants receiving bLfcin supplementation and the placebo group (29% vs. 31%, RR 0.95, 95% CI 0.86–1.04). Of particular note, the study found a low prevalence of invasive candidiasis with a total of only five episodes (0.3% vs. 0.2%), which aligns with UK population surveillance data. In a systematic review of ten RCTs enrolling 3,679 preterm infants (<37 weeks gestation), supplementation with Lfcin, either alone or in combination with probiotics, significantly reduced the incidence of all types of LOS (RR 0.56, 95% CI 0.36–0.86). However, for fungal sepsis in particular, the reduction did not reach statistical significance (RR 0.27, 95% CI 0.08–1.00), especially in very premature infants (RR 0.30, 95% CI 0.04–2.23) (168). In contrast, in another systematic review of 12 RCTs including 5,452 preterm infants, Pammi et al. found that Lfcin supplementation significantly reduced the incidence of LOS (RR 0.80, 95% CI 0.72–0.89) compared to placebo. The same effect was observed for fungal sepsis, both with Lfcin alone (RR 0.23, 95% CI 0.10–0.54) and in combination with probiotics (RR 0.24, 95% CI 0.08–0.71) (169).

According to the IDSA guidelines, oral bLfcin at a dose of 100 mg per day may be effective in VLBW infants, but the recommendation is graded as weak (12). The ESCMID guideline gives a moderate recommendation for daily treatment with 100 mg Lfcin, either as monotherapy or in combination with 10^6^ CFU *Lactobacillus*, starting on the third day of life and continuing until the sixth week of life or until discharge from NICU, to reduce the risk of fungal infections (133). Alternatively, many studies have shown that birth weight-dependent doses of 150–300 mg/kg/day are considered optimal (162, 169, 170).

## Clinical and neurodevelopmental outcomes

ICIs can affect multiple organs and tissues throughout the body, such as the brain, heart, kidneys, eyes, liver, spleen, lungs, bones, etc (171). The central nervous system (CNS) is often damaged, with meningitis being the most common form (172). Of particular note, this population usually has high mortality and subsequent severe NDI regardless of adequate antifungal treatment (109, 127), and this proportion is higher than in those infected with bacterial pathogens. There is an inverse relationship between birth weight or gestational age and the mortality rate of infants with ICIs, and this rate can be as high as 50% in ELBW infants (173). One study reported that infants with *Candida* infection had the highest risk of death and/or NDI among preterm infants with late-onset infection (174). Similarly, according to the study by Benjamin DK et al., among ELBW infants, those who developed candidiasis had the highest rate of NDI at 57%, while the rate among infants with bacterial infections or no infections was relatively low at 36% (86). Even more, nearly 70% of ELBW (birth weight <750 g) infants are reported to either die from or experience severe NDI following ICIs, despite treatment (127).

## Treatment and recommendations

Prevention of infection should be the most important goal in newborn infants, as it is a severe threat to this vulnerable population, who are at high risk of infection. However, if an infection is suspected or confirmed, prompt and appropriate treatment has been shown to reduce morbidity and mortality (109). Infections in infants tend to spread to multiple critical organs. In general, blood and urine cultures should be taken. If neonates have positive *Candida* spp. cultures from blood and/or urine, a lumbar puncture, and a dilated retinal examination are recommended to further assess for potential dissemination to the CNS and retina. In instances where *Candida* blood cultures remain persistently positive, disseminated ICIs should be suspected, and an ultrasound evaluation of the urogenital tract, liver, and spleen should also be performed (12). When selecting antifungal agents, it is important to determine whether the infection involves the CNS or the urinary tract. Removal of all foreign bodies, such as tubes, shunts, implants, or catheters, should be considered if possible, as *Candida* spp. tend to form biofilms on most materials, which are difficult to penetrate and hinder the effective eradication of the infection (18).

Timely initiation of empiric antifungal therapy is critical to treatment success, especially considering that it takes an average of 36 h for cultures to become positive, or up to 42 h if the infant is receiving antifungal prophylaxis (175). Empiric antifungal therapy has been shown to increase the survival rate without NDI in infants with ICIs (176). In addition to timely pharmacologic intervention, optimal antifungal agent selection and appropriate dosing are also important. When selecting agents for ICIs, clinicians should consider the predominant isolates of *Candida* spp. in their NICUs, *Candida* antifungal resistance patterns, and whether the infant has received prior antifungal prophylaxis, as well as the specific prophylactic agents. The current major antifungal agents for the treatment of neonates with ICIs are azoles, polyenes, and echinocandins (Table 6).

**Table 6:** The common antifungal agents for treating invasive *Candida* infections (ICIs) in neonate**s**.

Agents	Recommended dosing (Reference)	Major concerns in using	Potential mechanisms of action	Evidence on efficacy
Fluconazole	Loading dose 25 mg/kg on day one, then 12 mg/kg daily (178)	Hepatotoxicity	Disrupts the production of ergosterol, a fungal cell wall component, causing toxic lanosterol accumulation in the membrane	Moderate-quality evidence
Amphotericin B deoxycholate (AmB-D)	0.7–1 mg/kg daily (178)	Nephrotoxicity, moderate	Binds to ergosterol, then disrupts fungal cell wall synthesis, which leads to leakage of cellular components	Moderate-quality evidence
Lipid formulation amphotericin B (LFAmB)	3–5 mg/kg daily (12)	Nephrotoxicity, mild	Binds to ergosterol, then disrupts fungal cell wall synthesis, which leads to leakage of cellular components	Moderate-quality evidence
Micafungin	Loading dose 25 mg/kg on day one, then 10 mg/kg daily (178)	Hepatotoxicity; Potential risk of hepatic tumours if high dose, prolonged exposure	Inhibits 1,3-β-D-glucan synthase, an essential component of the fungal cell wall, which leads to cell lysis and eventually death	Moderate-quality evidence

Fluconazole, an azole antifungal agent, is the most studied agent for the prevention and treatment of fungal infections in neonates due to its favorable nephrotoxicity profile. It has high antifungal activity against *Candida* spp. and penetrates well into the CSF, making it ideal for CNS infections. It is mainly excreted via the kidneys and reaches high concentrations in the urine, which is beneficial in the treatment of *Candida* infections of the urinary tract. According to IDSA guidelines, fluconazole treatment at 12 mg/kg daily, either intravenously or orally, is a reasonable alternative for infants without previous fluconazole prophylaxis (12). Concurrently, fluconazole treatment should be avoided in infants if *Candida glabrata* or *Candida krusei* infection is suspected or confirmed. A loading dose of fluconazole at 25 mg/kg has demonstrated a more rapid achievement of target concentrations and does not increase the risk of hepatotoxicity, a rare adverse effect linked to fluconazole (177, 178).

Amphotericin B (AmB), a polyene antifungal agent, includes AmB deoxycholate (AmB-D) and lipid formulation AmB (LFAmB). It targets fungal cell wall synthesis by binding to ergosterol, triggering pore formation and subsequent leakage of intracellular components, and eventually presents fungicidal activity against susceptible organisms (179). Of the available AmB formulations, AmB-D is currently the major option for ICIs in neonates, with a recommended dose of 1 mg/kg daily (12, 178). Infusion-related reactions to this agent are virtually absent in neonates, and the toxicity risk in neonates is considered low, even though increased serum creatinine and hypokalemia have been observed (180). LFAmB is considered to have less renal toxicity due to its renal protective properties, as lower concentrations of the lipid formulation are present in the urinary tract. Therefore, LFAmB can be considered at a dose of 3–5 mg/kg daily when there is no urinary tract involvement in neonates (12).

Echinocandins are attractive agents given their efficacy against *Candida* biofilms and their extended spectrum against *Candida* spp., including often-resistant species such as *Candida parapsilosis*, *Candida glabrata*, *Candida krusei*, and others (181). The IDSA guidelines recommend that echinocandins should be used as a salvage therapy or in cases where resistance or toxicity precludes the use of AmB-D or fluconazole (12). Of particular note, echinocandins fail to attain therapeutic concentrations in the urine (182), rendering them unsuitable for the treatment of urinary tract infections. Furthermore, echinocandins do not penetrate the CSF, but they have demonstrated the ability to reach the brain parenchyma (183) and have proven effective in the treatment of *Candida* meningoencephalitis in neonatal animal models (184). Of the echinocandins, micafungin is the most preferred agent because of its efficacy and safety in the neonatal population, despite the observation of micafungin treatment discontinuation due to abnormal liver tests in multicenter trials (185). The mechanism of action involves the inhibition of β- (1, 3)-D-glucan synthesis, a critical component of the fungal cell wall, resulting in cell lysis and subsequent cell death (186). The recommended dosing of micafungin is a loading dose of 15 mg/kg on day one, then 10 mg/kg daily (178). We should be aware that micafungin carries a black box warning due to its association with hepatic tumors observed in murine models under conditions of high doses and prolonged exposure; thus, long-term safety remains in need of careful consideration in this vulnerable group of neonates. However, to date, such conditions and effects have not been observed in human subjects.

Current first-line agent recommendations include AmB-D or fluconazole (if the isolate is susceptible), with second-line and salvage therapies, including LFAmB and micafungin (12). The duration of antifungal therapy should continue for at least 2 weeks after documented clearance, which must be confirmed by microbiologic clearance, and in the absence of signs or symptoms indicating persistent infection (12). In the first week of therapy, the aim is to attain microbiologic clearance of the infection, which includes securing at least two blood cultures negative ≥24 h apart, as well as a negative urine culture, and a CSF culture (13). If microbiologic clearance does not occur during the first week, an alternative antifungal agent should be considered, such as micafungin, and source control must also be reassessed. In addition, special attention should be paid to the fact that a *Candida* infection of the urinary tract in premature infants, also known as “Candiduria”, generally means a disseminated *Candida* infection and is usually associated with a substantial risk of death or NDI (182). Positive urine cultures obtained from either sterile urethral catheterization or suprapubic aspiration should be considered equivalent to positive blood cultures. Meanwhile, this population should undergo a systemic workup (abdominal ultrasound, blood, and CSF cultures) for disseminated *Candida* infection that warrants treatment (12).

## Conclusions and future perspectives

ICIs continue to be a major clinical challenge for hospitalized newborn infants receiving intensive care, primarily affecting very premature and VLBW infants in HICs, whereas larger infants are more commonly affected in LMICs. The implementation of a series of proactive prevention and control strategies, such as the expanded use of prophylactic fluconazole, the prudent reduction of broad-spectrum antibiotics, and the increased adoption of empiric antifungal therapies, has contributed to a gradual decline in the incidence of ICIs in HICs. However, this problem is also a serious threat in LMICs, where limited access to these interventions and non-judicious use of broad-spectrum antibiotics remain key barriers. When ICIs are suspected, timely initiation of empirical antifungal therapy, along with prompt removal of compromised catheters, has been shown to improve clinical outcomes of neonates. Certainly, evidence-based prophylactic measures are critical for reducing the burden of ICIs in hospitalized newborn infants. Future efforts should prioritize standardized protocols for evidence-based prevention across intensive care units and track the trends of ICIs, particularly in LMICs with limited data and a large number of “outborn” newborns.
